# An Integrated Field-Effect Microdevice for Monitoring Membrane Transport in *Xenopus laevis* Oocytes via Lateral Proton Diffusion

**DOI:** 10.1371/journal.pone.0039238

**Published:** 2012-07-05

**Authors:** Daniel Felix Schaffhauser, Monica Patti, Tatsuro Goda, Yuji Miyahara, Ian Cameron Forster, Petra Stephanie Dittrich

**Affiliations:** 1 Department of Chemistry and Applied Biosciences, ETH Zurich, Zurich, Switzerland; 2 Institute for Physiology, University of Zurich, Zurich, Switzerland; 3 Institute of Biomaterials and Bioengineering, Tokyo Medical and Dental University, Tokyo, Japan; Dalhousie University, Canada

## Abstract

An integrated microdevice for measuring proton-dependent membrane activity at the surface of *Xenopus laevis* oocytes is presented. By establishing a stable contact between the oocyte vitelline membrane and an ion-sensitive field-effect (ISFET) sensor inside a microperfusion channel, changes in surface pH that are hypothesized to result from facilitated proton lateral diffusion along the membrane were detected. The solute diffusion barrier created between the sensor and the active membrane area allowed detection of surface proton concentration free from interference of solutes in bulk solution. The proposed sensor mechanism was verified by heterologously expressing membrane transport proteins and recording changes in surface pH during application of the specific substrates. Experiments conducted on two families of phosphate-sodium cotransporters (SLC20 & SLC34) demonstrated that it is possible to detect phosphate transport for both electrogenic and electroneutral isoforms and distinguish between transport of different phosphate species. Furthermore, the transport activity of the proton/amino acid cotransporter PAT1 assayed using conventional whole cell electrophysiology correlated well with changes in surface pH, confirming the ability of the system to detect activity proportional to expression level.

## Introduction

For many decades, electrophysiological methods have been at the forefront of investigations of membrane conduction and excitation in living cells. [1] To measure membrane conductance, control of either the transmembrane voltage or current is required. Therefore, a key experimental challenge is to establish adequate electrical access to the cytosol. For example, in the commonly used two electrode voltage clamp (TEVC) applied to large cells such as *Xenopus laevis* oocytes, two microelectrodes impale the oocyte to sense and control the membrane potential. This procedure requires delicate glass microelectrodes and a high degree of micromanipulation, either by a human operator or precision robotics. [2], [3]. In the whole cell patch clamp, electrical access to the cytosol is gained by sealing the tip of a glass microelectrode to the membrane and applying suction to eventually rupture the cell membrane within the electrode. [4] Recently, several non-invasive voltage clamp techniques for *Xenopus laevis* oocytes have been developed that leave the membrane intact. These methods rely on the physical compartmentalization of the membrane into two areas and measurement of trans-cellular currents across the entire oocyte. In the transoocyte voltage clamp (TOVC) an AC voltage is applied across symmetrically distributed membrane impedances. [5] Asymmetric variants of the TOVC have been realized to better define the transmembrane potential across a smaller region of membrane [6], [7] and are akin to the loose macropatch. [8].

Even though voltage clamping has established itself as a reliable tool for performing electrophysiological experiments, its application in large-scale industrial screening has not been fully realized. Apart from the aforementioned cell manipulation requirements, parallelization of the voltage clamp requires duplication of many hardware components - including the electrodes, voltage clamp electronics and the fluidic pathways. Electronic detection methods based on field-effect devices, on the other hand, are attractive alternatives for large-scale integration. As the sensors detect local surface charge accumulation or depletion as opposed to passing current, from the cell medium, liquid domains can be shared without significant crosstalk occurring between channels. Furthermore, as the sensing field is limited by the shielding properties of ionic solutions, this would allow the realization of high density sensor arrays. The detection distance can be calculated using Debye’s relation for low electrolyte concentrations. [9] Recent work on the theory of electrostatic fields in ionic solutions allows for more accurate predictions on high electrolyte concentrations, as present in physiological solutions. [10] The most appropriate field-effect sensor for use in ionic solutions, the ion-sensitive field-effect transistor (ISFET), has first been described by Bergveld. [11] The ISFET, which is derived from the metal-oxide field-effect transistor (MOSFET), replaces the metal gate electrode on the insulator with the bulk solution, the potential of which is defined using a reference electrode. Charge separation at the insulator-liquid interface then results in a detectable potential difference due to the double-layer capacitance between the reference electrode and the semiconducting channel. ISFETs have mostly been used to measure local changes in pH, such as acidification in cell cultures. [12], [13] There has also been interest in using ISFETs for studying membrane transport. The first successful transport assays were reported on the human anionic transporting peptide C (OATP-C) heterologously expressed in *Xenopus laevis* oocytes. [14] In these experiments, the uptake of estrone-3-sulfate and estradiol 17β-D-glucuronide was detected with good sensitivity, and differences in uptake rates between the wild-type and mutant of the OATP-C transporter were resolved.

*Xenopus laevis* oocytes, like other cells, are believed to have endogenous proton-regulating mechanisms at the cell membrane that are responsible for a pH gradient across the cell membrane. In particular, there is evidence of the existence of a Na^+^/H^+^ exchanger that is at least partially responsible for the proton countertransport across the cell membrane. [15], [16] Furthermore, overexpression of proton-dependent membrane proteins modulates local proton binding affinity upon transport activation. Transport activity mediated by heterologously expressed transport systems by pH detection using a glass microelectrode placed in close proximity to the membrane has been demonstrated. [17], [18] Considering the extremely fast bulk diffusion rate of protons in water, [19], [20] it may at first seem surprising that pH values different from the bulk solution can be detected at all. However, proton sinks and sources at the cell membrane change the local association/dissociation rate constants that results in lateral diffusion rates different from the bulk diffusion rate. Recently, a mathematical model for lateral diffusion kinetics at the cell membrane for protons was described, [21] which provides numerical solutions for the dwell time of a proton in buffered solutions commonly used in physiology. The results support previously conducted experiments that show that a proton can migrate laterally hundreds of micrometers along the cell membrane before diffusing back into the bulk solution. [22].

**Figure 1:**
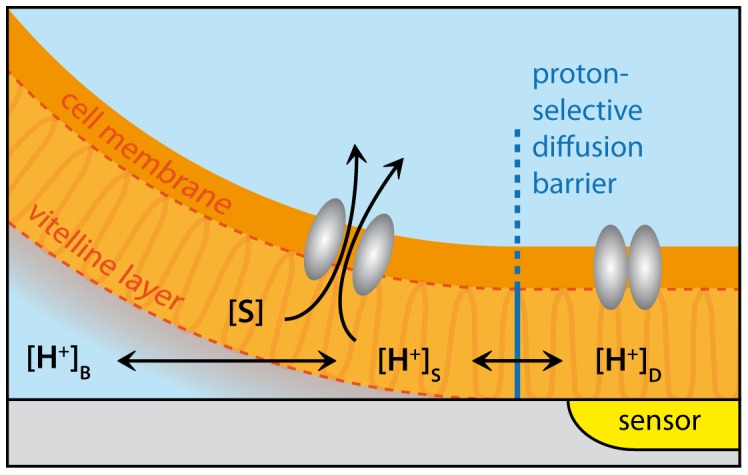
Lateral proton diffusion model. Simplified cartoon of the proton-selective lateral diffusion model where the oocyte interfaces to the sensor element (not to scale). The vitelline layer of the (defolliculated) oocyte comprises a network of fibrous filaments that surround the cytoplasmic membrane, which itself is not smooth due to numerous microvilli protrusions (not shown). The total thickness of the vitelline layer is estimated to be 2–3 µm for stage VI oocytes. [39] ([S]: concentration of substrate interacting with the membrane transport proteins. [H^+^]_D_, [H^+^]_S_, [H^+^]_B_: Proton concentration at the detection site, membrane surface and in buffer, respectively.) We assume the diffusion barrier is narrow compared with the sensor area so that “edge effects” can be neglected and transporters within the sensor region do not “see” the substrate S.

Based on the lateral diffusion model, we have developed a method for sensing proton-dependent membrane transport in *Xenopus laevis* oocytes by utilizing a novel arrangement of ISFET technology. We establish a proton-selective diffusion barrier that is created by direct contact of part of the membrane with the sensor. The membrane is stabilized against the sensor surface using our previously reported immobilization technology. [6] Due to the much higher lateral diffusion rate of the protons compared to other solutes, the detected potential change at the sensor is exclusively proton-dependent (Fig. 1). Once equilibrated, the proton concentration at the detection site [H^+^]_D_ is equal to the proton concentration at the membrane surface [H^+^]_S_, assuming ideal coupling between the sensor surface and the membrane surface. The surface potential of the sensor then reflects the surface pH for steady state membrane transport. We predict that in contrast to protons, substrate molecules *S* that interact with transport proteins at the cell membrane exposed to the bulk solution, will diffuse poorly across the diffusion barrier for two reasons. First, their lateral mobility relative to the protons is greatly reduced and second, they are effectively removed from the local medium by the transport proteins themselves before reaching the detection surface. As a consequence, modulation of substrate concentration in proximity to the detection surface will be minimal for large migration distances, as present in our system.

To verify the validity of the proposed method, we conducted experiments on oocytes overexpressing the electrogenic sodium/phosphate cotransporters PiT-2 (SLC20A2) and NaPi-IIb (SLC34A2), the electroneutral isoform NaPi-IIc (SLC34A3) and the proton/amino acid cotransporter PAT1 (SLC36A1). The goal of this work is to demonstrate that our arrangement allows for non-invasive assays of transport processes, which result in local pH changes under physiological conditions.

## Materials and Mehods

### System Description

**Figure 2:**
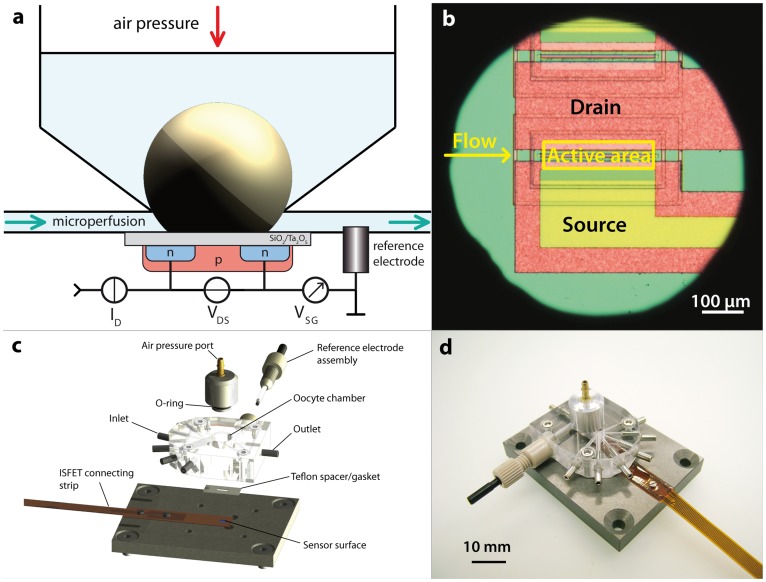
Design of the microdevice. a) Schematic view of the cross-section of the device (not drawn to scale). The height of the microperfusion channel is around 200 µm. A typical Xenopus oocyte would be 1000–1200 µm in diameter. b) Micrograph of the sensor as seen through the hole (ca. 800 µm in diameter) of the oocyte immobilization compartment. The oocyte membrane completely covers the active area of the sensor due to its deformability. c) Exploded view of the device using the original 3D CAD engineering data. d) Photograph of the assembled, but unconnected device.

The device comprises a sensing element, a microperfusion system and a circular orifice for immobilization of the oocyte and alignment with the sensing surface (Fig. 2). The sensor is an n-channel field-effect transistor (FET) without metal gate and with a 40 nm thick layer of tantalum pentoxide (Ta_2_O_5_) as the insulator material (ISFETCOM Co. Ltd., Saitama, Japan). Ta_2_O_5_ exhibits superior proton buffering capacity compared to other metal oxides while providing a good diffusion barrier. [23] The sensor was inserted into the cavity of a precisely machined support made of aluminum, creating a flush fit. A rectangular (5 mm×10 mm) piece of perfluoroethylene (PTFE) with a thickness of 0.2 mm was then placed over the sensor to serve as a gasket and spacer for forming the microperfusion channel. A CNC-machined block made from poly(methyl methacrylate) (PMMA) was screwed onto the aluminum support. It integrated 6 liquid inlet channels and 1 outlet channel as well as the immobilization orifice. A hole (1.6 mm dia.) intersecting with the outlet channel was drilled for insertion of the reference electrode. Due to the low dimensional tolerances of all parts involved, the oocyte orifice is self-aligned with the sensor surface upon assembly of the device.

The FET was driven with a source-drain follower circuit that fixed the source-drain voltage and drain current to constant values. For all experiments, the source-drain voltage and current were set to 500 mV and 500 µA, respectively. The reference electrode was connected to the circuit’s signal ground which defined the reference potential, against which the output signal *V_SG_* was measured at the source connection. In this configuration, changes in *V_SG_* are directly proportional to changes in the Nernst potential. The technical implementation of the drain-source follower was realized using constant current drivers. [24] Acquisition of the gate-source voltage was achieved using a high-resolution data acquisition and control (DAQ) unit (LabJack U6-Pro, LabJack Corp./USA). The DAQ unit also served as a controller for the air pressure system used for cell immobilization and solution exchange. User control of all systems was provided by our proprietary PC-based graphical user interface (GUI), which employs the Windows.NET application programming interface (Microsoft Corp./USA). An expanded version of an air pressure system, which provided solution exchange and perfusion, as well as oocyte immobilization was used as described earlier. [6].

### Solutions and Reagents

For storage of oocytes, modified Barth’s solution contained (in mM): 88 NaCl, 1 KCl, 0.41 CaCl_2_, 0.82 MgSO_4_, 2.5 NaHCO_3_, 2 Ca(NO_3_)_2_, 7.5 HEPES, pH was adjusted to 7.5 with Tris and supplemented with 5 mg/l doxycycline and 5 mg/l gentamicine. Standard buffered saline solutions (100 Na) contained (in mM): 100 NaCl, 2 KCl, 1 MgCl_2_, 1.8 CaCl_2_, 10 HEPES, adjusted to pH 7.5, pH 7.4 or pH 6.5 with TRIS. For test solutions containing activating or inhibiting substances the following stock solutions were used: 1 M (in H_2_O) inorganic phosphate (P_i_) at pH 7.4, 100 mM (in DMSO) amiloride, 100 mM (in H_2_O) L-Proline, 100 mM (in H_2_O) γ-Aminobutyric acid (GABA) and 100 mM (in H_2_O) sodium phosphonoformate tribasic hexahydrate (PFA). After addition of the reagents, all solutions were filtered using a 0.22 µm filter, prior to filling the solution vials. All standard reagents were purchased from either Sigma-Aldrich or Fluka.

### Oocyte Preparation

Female *Xenopus laevis* frogs were purchased from Xenopus Express (France) or African Xenopus Facility (R. South Africa). The frogs were anesthetized in MS222 (tricaine methanesulphonate) after which portions of ovaries were surgically removed and cut in small pieces. Collagenase treatment on oocytes was done for 45 min with 1 mg/ml of crude type 1A in 100 Na solution (without Ca^2+^) in presence of 0.1 mg/ml trypsin inhibitor type III-O. Healthy stage V-VI oocytes were selected, maintained in modified Barth’s solution at 16°C and injected with typically 5 ng total of cRNA of the α,β,γ sub-units of the *Xenopus* isoform of ENaC, [25] GAT1, [26] flounder NaPi-IIb, [27] mouse NaPi-IIc, [28] mouse PAT1 [29] or human PiT-2 [18] according to procedures previously conducted in our laboratory. After injection, the oocytes were incubated for 3 days at 16°C in Barth’s solution. Non-injected oocytes of the same batch were used as negative controls. All animal handling procedures were approved by Swiss Cantonal and Federal veterinary authorities.

### Procedure for Oocyte Experiments

Before the experiment, the oocytes were thoroughly washed in 100 Na solution at pH 7.4 to prevent the diffusion of protons between the oocyte chamber and the microperfusion channel. After priming of the system with 100 Na pH 7.4 solution, the cell was pipetted into the oocyte chamber. The air port plug was then inserted into the oocyte chamber and a constant air pressure of 5 mbar was applied. The perfusion system was then pressurized at 100 mbar which, in combination with the inlet capillaries (30 cm length, 200 µm inner diameter), resulted in a flow rate of approx. 1 µl/s when one of the solenoid valves was open. The oocyte was perfused with 100 Na solution at pH 6.5 (for PAT1) or pH 7.4 (all others) for 5 min to create stable steady-state conditions before initiating a transport activation protocol. The perfusion sequence was programmed beforehand using a built-in macro feature of the GUI. This feature provided high timing accuracy of the solution switching and guaranteed the repeatability of the perfusion sequence.

## Results and Discussion

### pH-sensing Characterization of the ISFET

**Figure 3:**
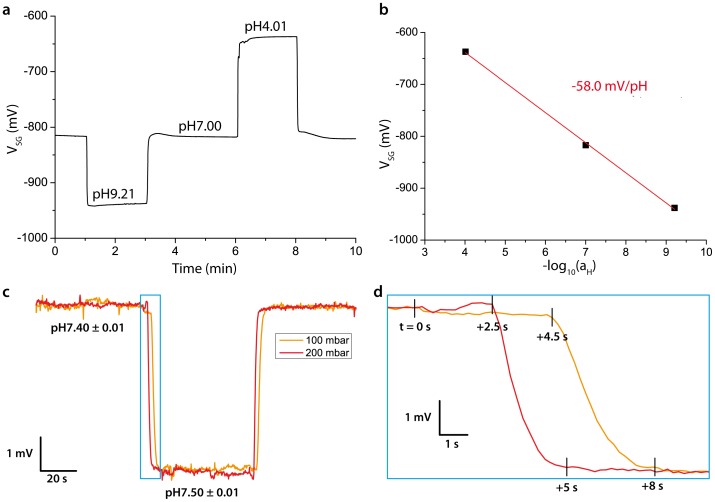
pH reference measurements. a) Time-dependent measurement of phosphate reference buffers at pH 4.01, pH 7.00 and pH 9.21 and b) linear fit of the data points obtained from the baselines of each buffer. Slope and standard error of the linear fit are shown. c) Time-dependent measurement of 100 Na solutions buffered at pH 7.40 and pH 7.50 for two different perfusion pressures (100 mbar and 200 mbar). d) Expanded view of data in panel c during the exchange of solutions from pH 7.40 to pH 7.50. Valves were switched at t = 0 s.

To determine the response of the sensor to changes in bulk proton concentration and correlate the changes in output voltage to the pH change, the sensor was superfused with buffered solutions at varying pH. In a representative measurement, a slope of −58.0 with a linearity error of 1.5 mV/pH (R_adj_^2^ = 0.99862) was found using a three-point extrapolation with standard phosphate buffer solutions at pH 4.01, pH 7.00 and pH 9.21 (Fig. 3a,b). This value was close to the prediction from the Nernstian equation (−59 mV/pH), which demonstrated the excellent proton buffering capacity of Ta_2_O_5_. The detection limit of ΔpH was determined to be approx. 0.005 units (signal-to-noise ratio of 3). The drift slope depended on a number of factors, such as ambient light intensity, but was typically less than 0.1 mV/min. The sensor-to-sensor variation was insignificant, which resulted from the large sensor structure and precise CMOS manufacturing processes. In a second experiment, the sensor was superfused with 100 Na solutions at pH 7.50 and pH 7.40 (Fig. 3c). The experiment showed an expected decrease of 5.4 mV (i.e. ΔpH = −0.1) with a rapid transition from the first steady-state signal to the next one. Even though the flow inside the microperfusion channel was laminar, the signal response was not perfect due to cross-diffusion at the junction where the inlet channels meet. Also, there was some dead time between the switching of the valves and the signal response onset due to the relatively large solution exchange volume (approx. 4 µl) in relation to the flow rate (1 or 2 µl/s). In total, it took approx. 8 s at 100 mbar and approx. 5 s at 200 mbar to reach steady-state conditions after valve actuation, demonstrating that the sensor response was predominantly limited by the flow rate (Fig. 3d). Nevertheless, for the uptake experiments on oocytes these values are fully acceptable due to the comparatively slow proton diffusion between the membrane surface and the sensor surface (see below). With regards to selectivity, the sensitivity of the Ta_2_O_5_-based sensor towards Na^+^ and K^+^ is less than 1 mV/M, as has been described previously. [30].

### Transport Experiments on Oocytes Heterologously Expressing Various Membrane Transport Proteins

#### Proline transport mediated by PAT1

**Figure 4:**
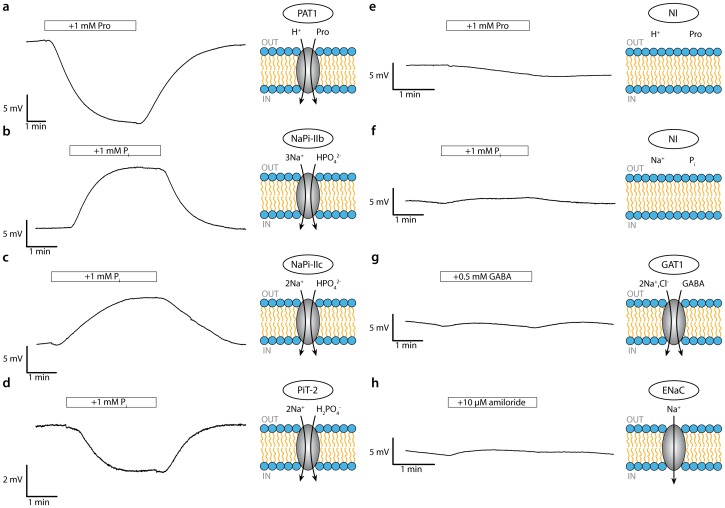
Membrane transport experiments. Experiments conducted on oocytes heterologously expressing various membrane transport proteins indicated with their respective controls on non-injected (NI) oocytes showing sensor readout (V_SG_) as a function of time. Only part of the initial stabilizing baseline region that preceded substrate application is shown (see Materials and Methods): a) PAT1, b) NaPi IIb, c) NaPi-IIc, d) PiT-2, e) Proline control, f) P_i_ control, g) GAT1, h) ENaC. In each case either the same or representative oocytes from the same batch were pretested using a two-electrode voltage clamp to confirm functional expression. The bars indicate the duration of application of the respective activating and blocking agents. Arrows indicate flux direction of substrate according to the assumed driving force conditions.

Assays conducted on oocytes heterologously expressing the proton-driven amino acid transporter (PAT1) showed a significant decrease of *V_SG_* upon exposure of the cell to a 1 mM proline solution (Fig. 4a). The signal reached a steady state after 5 min and returned to the initial baseline after washout of proline. PAT1 is known to reversibly bind amino acids and cotransport them stoichiometrically with one proton/amino acid per cycle. [31] Under our experimental conditions, we attribute the downward deflection of *V_SG_*, upon exposure of the cell to proline, to reflect a lateral movement of protons away from the sensor region due to the depletion of protons at the extracellular membrane surface during the H^+^/proline coupled transport. Moreover, given the large volume of the oocyte and low transport turnover rate of PAT1, we would expect neither the intracellular proline concentration nor intracellular pH to change sufficiently during the experiment such that reverse mode behavior (proton and proline efflux) might occur in the region of oocyte membrane exposed to the sensor. Thus, even though PAT1 works bidirectionally, the exposure time of 5 minutes was apparently insufficient to significantly change the proton gradient across the cell membrane.

#### Phosphate transport mediated by NaPi-IIb and PiT-2

In contrast to PAT1, for oocytes heterologously expressing the electrogenic sodium-coupled phosphate cotransporter (NaPi-IIb) the signal deflection was reversed, which indicated that a decrease in surface pH occurred (Fig. 4b). This can be understood when we consider that P_i_ is present at the membrane surface as both divalent (HPO_4_^2−^) and monovalent (H_2_PO_4_^−^) phosphate species with an assumed pK_a_ = 6.8 under physiological conditions and distributed according to the following equilibrium,
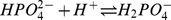
(1.1)The electrogenic NaPi-IIb translocates 3 Na^+^ together with one divalent P_i_ per transport cycle, [32] which results in the increase of [H^+^]_S_ due to the depletion of the divalent species.

For the electroneutral isoform NaPi-IIc, a detectable pH decrease at the membrane surface is also induced upon exposure to P_i_ (Fig. 4c). This was expected given that NaPi-IIc also prefers divalent P_i_, [33] and translocates two Na^+^ per cycle with no net charge movement. [34] This result also establishes that the sensor (i) is capable of sensing electroneutral transport processes and (ii) does not simply respond to a change in the cell membrane potential as might be predicted for the electrogenic transporters that mediate net charge translocation.

In contrast to the SLC34 family of sodium coupled P_i_ cotransporters, members of the SLC20 family prefer monovalent P_i_ (H_2_PO_4_^−^), and translocate two Na^+^ per transport cycle together with one positive net charge. The resulting shift of the equilibrium to the right (see equation above) would therefore result in a decrease in [H^+^]_S_, which explains the downward deflection (increase in pH) of *V_SG_* in PiT-2 upon exposure to P_i_ (Fig. 4d). The amplitude of the change in *V_SG_* at steady state was significantly lower compared to NaPi-IIb and NaPi-IIc and most likely resulted from the lower surface expression of protein compared to NaPi-IIb,c. The changes in surface pH registered by the ISFET sensor for NaPi-IIb and PiT-2 were in qualitative agreement with TEVC measurements conducted in combination with surface pH measurements using a pH-sensitive glass microelectrode. [18].

#### Control experiments confirm the specificity of the sensor for detecting local ΔpH

Control experiments on non-injected oocytes showed minimal deflections of *V_SG_*, both for P_i_ and proline (Fig. 4e,f). These may be due to endogenous membrane proteins that could interact with proline and P_i_. In particular, the presence of amino acid-modulated membrane activity has been determined in uptake studies. [35] Nonetheless, considering that relatively high concentrations of proline and P_i_ were used in our experiments, the contribution of the endogenous activity can be neglected when the level of exogenous expression is sufficiently high. To further demonstrate that modulation of proton-independent transport proteins did not induce a potential change at the sensor surface, we conducted experiments on oocytes overexpressing the γ-aminobutyric acid (GABA) transporter (GAT1) (Fig. 4g) and the epithelial sodium channel (ENaC) (Fig. 4h). GAT1 is a sodium-chloride-dependent cotransporter highly specific for GABA. [36] ENaC is a sodium channel whose conductance can be blocked using amiloride (K_i_<1 µM). [37], [38] For oocytes expressing either of these membrane proteins, we were unable to resolve a significant change in *V_SG_*. This finding allowed us to exclude the presence of detection artifacts at the sensor surface and confirmed the specificity of the sensor to changes in local proton concentration.

#### Correlating the ISFET response to transport activity

**Figure 5:**
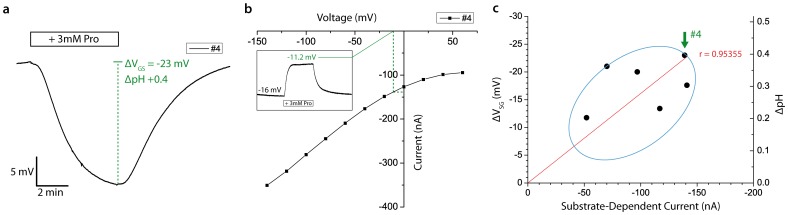
Correlation of pH response with protein expression level. Correlating sensor response with transport activity. a) Sensor response to proline superfusion of a representative oocyte (designated #4 in c) heterologously expressing PAT1. b) TEVC I-V data of the proline-dependent current of oocyte #4 in response to the addition of 3 mM proline solution to the 100 Na buffer. Inset shows the change in membrane potential induced by proline application for the same oocyte as in a. c) Correlation of ΔV_SG_ and the substrate-dependent current. Each point represents data from a single oocyte. Arrow marks the data point of oocyte #4 (−23 mV, −140 nA).

We used oocytes overexpressing PAT1 to correlate the change in membrane surface pH with PAT1 activity. For the oocyte on the ISFET sensor, application of proline will result in a net intracellular translocation of protons into the oocyte, and a concomitant membrane depolarization. A new steady-state membrane potential will be reached that is a function of both the PAT1 protein expression level and transport rate, as well as the endogenous leak conductance of the oocyte. The latter effectively shunts the secondary active transport process mediated by PAT1. As the endogenous leak may vary from cell to cell, for a given expression level, the change in membrane potential will also vary and is therefore not a good quantitative measure of transport activity. Moreover, the proline transport rate will itself be a function of membrane potential due to voltage-dependent partial reactions in the transport cycle. [29] Therefore, to correlate transport activity with ΔpH detected by the sensor for individual oocytes (Fig. 5a), we first determined the steady-state membrane potential reached during transport under the same conditions as for the ISFET assay (inset, Fig. 5b). For that case, the net membrane current is zero and comprises the inward transporter-related flux that is balanced by an equal, but opposite current mediated by endogenous channels and pumps. We then voltage clamped the oocyte to determine the transporter-related current by subtracting the current in the absence of proline from that obtained with proline to eliminate proline-independent endogenous currents. From this I-V data we could estimate the transport flux corresponding to the steady-state potential for the ISFET assay (Fig. 5b). To ensure minimal substrate accumulation, we first tested the response using the TEVC to avoid long substrate exposure incurred with the ISFET system.

We observed a reasonable correlation between changes in *V_SG_* and the substrate-dependent current for six oocytes expressing PAT1 (Fig. 5c). This finding supports the assumption that the change in surface proton concentration is directly proportional to the total substrate current, which for a given turnover rate and driving force, is a measure of the number of active transporters in the membrane. Indeed, this is to be expected if the dependence of protons on the translocation of a substrate-specific substance is stoichiometric. The substrate turnover rate is then proportional to [H^+^]_S_, which allows quantitative substrate activation and inhibition studies. This implies that a high coupling strength between [H^+^]_S_ and [H^+^]_D_ is maintained to minimize secondary effects compromising the linearity of concentration change. We assume that optimizing the geometry of the structures involved in the immobilization of oocytes (hole diameter, sensor-to-hole distance) will lead to improved coupling strength between the membrane transport proteins and the sensor surface, without compromising the effectiveness of the proton-selective diffusion barrier. This may also be the reason for the deviation of data points in Fig. 5c.

### Conclusion

We present an integrated microdevice based on ISFET technology for monitoring proton-dependent membrane transport. Based on the ability of protons to migrate along the cell surface without diffusing back into the bulk solution, our method can sense surface pH free from detection artifacts stemming from other solutes. To verify the hypothesis, we conducted experiments on *Xenopus laevis* oocytes heterologously expressing various membrane transport proteins. Only transport systems that resulted directly (e.g. PAT1) and indirectly (e.g. NaPi-IIb,c, PiT-2) in a change in local pH gave significant responses. The results show that surface pH can be monitored with high precision and reliability. Moreover, control experiments demonstrate that solutes involved in the transport cycle do not diffuse along the cell membrane with sufficient efficiency to be detected by the sensor. Furthermore, studies correlating the change in surface pH with the population density of the amino acid cotransporter PAT1 have been done, revealing the feasibility of quantitative experiments, such as dose-response screenings. Optimization of the coupling between the transporter surface and the detection site will then lead to devices suitable for large-scale integration with potential for efficient and cost-effective high-throughput screenings. Furthermore, application to other cell types may be feasible by further miniaturization and modification of the cell immobilization site.
